# Endocrine Disruptors Acting on Estrogen and Androgen Pathways Cause Reproductive Disorders through Multiple Mechanisms: A Review

**DOI:** 10.3390/ijerph18041464

**Published:** 2021-02-04

**Authors:** Saira Amir, Syed Tahir Abbas Shah, Charalampos Mamoulakis, Anca Oana Docea, Olga-Ioanna Kalantzi, Athanasios Zachariou, Daniela Calina, Felix Carvalho, Nikolaos Sofikitis, Antonios Makrigiannakis, Aristidis Tsatsakis

**Affiliations:** 1 Department of Biosciences, COMSATS University Islamabad, Islamabad 44000, Pakistan; saira_amir@comsats.edu.pk (S.A.); syedtahirabbas@comsats.edu.pk (S.T.A.S.); 2 Department of Urology, University General Hospital of Heraklion, Medical School, University of Crete, 700 13 Heraklion, Greece; 3 Department of Toxicology, Faculty of Pharmacy, University of Medicine and Pharmacy, Petru Rares, 200349 Craiova, Romania; 4 Department of Environment, University of Aegean, University Hill, 81100 Mytilini, Greece; kalantzi@aegean.gr; 5 Department of Urology, Ioannina University School of Medicine, 45110 Ioannina, Greece; zahariou@otenet.gr (A.Z.); akrosnin@hotmail.com (N.S.); 6 Department of Clinical Pharmacy, University of Medicine and Pharmacy of Craiova, 200349 Craiova, Romania; calinadaniela@gmail.com; 7 UCIBIO, REQUIMTE, Laboratory of Toxicology, Faculty of Pharmacy, University of Porto, Rua de Jorge Viterbo Ferreira, 228, 4050-313 Porto, Portugal; felixdc@ff.up.pt; 8 Department of Obstetrics and Gynecology, Medical School, University of Crete, 71003 Heraklion, Greece; makrygia@uoc.gr; 9 Department of Forensic Sciences and Toxicology, Faculty of Medicine, University of Crete, 71003 Heraklion, Greece

**Keywords:** androgens, endocrine disrupting chemicals, estrogens, female, gonadal steroid hormones, human, infertility, male, mammals, reproduction

## Abstract

Increasing contamination of the environment by toxic compounds such as endocrine disrupting chemicals (EDCs) is one of the major causes of reproductive defects in both sexes. Estrogen/androgen pathways are of utmost importance in gonadal development, determination of secondary sex characteristics and gametogenesis. Most of the EDCs mediate their action through respective receptors and/or downstream signaling. The purpose of this review is to highlight the mechanism by which EDCs can trigger antagonistic or agonistic response, acting through estrogen/androgen receptors causing reproductive defects that lead to infertility. In vitro, in vivo and in silico studies focusing on the impact of EDCs on estrogen/androgen pathways and related proteins published in the last decade were considered for the review. PUBMED and PUBCHEM were used for literature search. EDCs can bind to estrogen receptors (ERα and ERβ) and androgen receptors or activate alternative receptors such as G protein-coupled receptors (GPCR), GPR30, estrogen-related receptor (ERRγ) to activate estrogen signaling via downstream kinases. Bisphenol A, dichlorodiphenyltrichloroethane, dichlorodiphenyldichloroethylene, polychlorinated biphenyls and phthalates are major toxicants that interfere with the normal estrogen/androgen pathways leading to infertility in both sexes through many ways, including DNA damage in spermatozoids, altered methylation pattern, histone modifications and miRNA expression.

## 1. Introduction

Any xenobiotic that can interfere with the normal secretion, production, metabolism, transport or effect of a hormone can lead to abnormalities in development, reproduction or homeostasis and hence be termed as an endocrine disrupting chemical (EDC) [1]. EDCs can be released in the environment in many forms. These include pesticides, industrial waste, packaging, synthetic food and cosmetics [2]. Over the last decades, mounting scientific evidence has shown that the reproductive health of humans and wildlife has been adversely affected by the EDCs [3,4,5,6,7].

Infertility is the inability of a sexually active, non-contraceptive couple to achieve spontaneous pregnancy within a year [8]. Primary infertility refers to couples that have never had a child and cannot achieve pregnancy after at least 12 consecutive months having sex without using birth control methods, while secondary infertility refers to infertile couples who have been able to achieve pregnancy at least once before [9]. One in eight and one in six couples encounter problems when attempting to conceive a first child and a subsequent child, respectively, while an overall rate of about 15% of couples fail to achieve pregnancy within one year and seek medical treatment [9]. Important treatment decisions are based on the results of semen analysis; therefore, the complete laboratory work-up should be standardized [9] according to the current World Health Organization (WHO) guidelines of modern semen analysis [10].

EDCs affect the reproductive system and the cellular processes in both sexes, leading to congenital abnormalities and infertility [11,12,13,14,15,16,17,18]. Mechanisms through which EDCs exert their pathophysiological effects have not yet been fully elucidated in human studies [19]. Despite a growing literature on couple exposure to non-persistent EDCs and fecundability, evidence for associations between biologically measured exposures and time to pregnancy is limited with equivocal results that do not preclude action, given the documented endocrine disrupting effects on other reproductive outcomes/fetal development [20]. Human exposure to persistent organic pollutants (POPs) such as polychlorinated biphenyles (PCBs) and dichlorodiphenyltrichloroethane (DDTs) is considered a cause of many disorders and can even be lethal [13,21,22]. Many animal and epidemiological studies confirm the detrimental effects of PCBs and DDT on the reproductive system [23,24]. It has been observed in recent years that fertility is declining at very high rates [25,26]. Decreased fertility rates in both men and women worldwide can be attributed to the increasing amounts of environmental toxicants [23,27,28].

The route of exposure to EDCs depends on the type of chemical and its use. It can be via food in the case of pesticides absorbed by plants, fish affected by contaminated water and drinking toxic water [29,30]. Some EDCs are components of synthetic food products like food coloring additives, while others migrate into food from packaging [31,32]. Air can also bring EDCs to the respiratory system as air pollutants such as waste smoke from factories, household dust and aerosol sprays (pesticides and insecticides) [33]. Contaminated water bodies contribute to a wider dispersal. Newborns can be exposed through breast milk from the mother. Another route of exposure is occupational exposure, namely when farmers do not use proper clothing while spraying the pesticides, enter the field after it has been sprayed or when empty pesticide containers are used for other purposes. Individuals working in the industries that synthesize or manipulate these chemicals can also be at higher risk of exposure than others. Despite the fact that the use and manufacturing of some of the chemicals such as PCBs and DDTs is banned throughout the world, they still persist in the environment as a result of excessive use in the past [34]. These compounds and their toxic metabolites persist in the environment due to their long half-lives and still pose a risk to living organisms [35,36]. Common-sense lifestyle changes in which both females and males seeking to conceive minimize exposure to non-persistent EDCs in consumer products have been advocated [20].

The two major pathways involved in the sexual development and process are androgen and estrogen pathways. EDCs affect both pathways leading to developmental and reproductive abnormalities in humans and animals. Several bioassays including in vivo, in vitro, and in silico studies have been used in past decades to confirm the health effects of these chemicals, namely the negative impacts of EDCs on the reproductive system and the significant exposure of various EDCs in infertile individuals [37,38].

## 2. Objective and Review Questions

The objective of the present comprehensive review is to discuss the broader targets of EDCs and the mechanistic pathways involved. The main aim is to collect the most recent data on EDCs involved in developmental and reproductive disorders. We address the way these compounds target the estrogen/androgen pathways and how they bind to the receptors to mediate their effects on the reproductive system. Furthermore, we address the ways in which these compounds cause abnormalities, i.e., whether they are genotoxic, cytotoxic or only cause epigenetic variations. EDCs affecting the synthesis, metabolism and release of endogenous estrogens/androgens are not addressed in the present study. The studies included are animal studies, cell culture and case-control epidemiological reports published during the last decade. For ligand binding mechanisms, in-silico studies were considered. Pubmed (http://www.ncbi.nlm.nih.gov/pubmed) and PubChem (https://pubchem.ncbi.nlm.nih.gov/) were used. Only four major classes of EDCs, namely bisphenol A (BPA), PCBs, DDT, dichlorodiphenyldichloroethylene (DDE) and phthalates were considered for inclusion, owing to their estrogenic and androgenic potential. The following keywords and their combinations were used: *endocrine disruptors, estrogen pathway, androgen pathway, BPA binding to ER, BPA binding to AR, PCB binding to ER, PCBs binding to AR, DDT binding to ER, DDT binding to AR, BPA and methylation, PCBs and methylation, DDT and methylation, polychlorinated diethyl ethers binding to ER and AR, polychlorinated diethyl ethers bind to ER and methylation, DDT in histone modifications, phthalates binding to ER and AR, phthalates in methylation, phthalates in histone modifications, DNA damage caused by EDCs, DNA damage and infertility, DNA damage and BPA*. Only English language studies specific to the estrogen or androgen pathway proteins were taken into consideration.

## 3. Results and Discussion

The first and most important event after exposure to EDCs is the binding of the chemical to the receptors to exert its action, affecting the normal functioning of the pathway. These can act either as agonists or antagonists. Almost all major categories of EDCs studied were found to bind to either androgen receptors (AR) and/or estrogen receptors (ER) (Table 1). Estrogens are a family of steroid hormones produced by different parts of the body including gonads, placenta, breasts, bones, adipose, vascular tissues and certain parts of the brain. Although estrogens are present in both males and females, their level of expression is variable. Their main functions include the development of secondary sex characteristics in females such as breast development. These are also involved in the thickening of the endometrium and the regulation of menstrual cycle. In men, these hormones are expressed in lower levels and control the spermatogenesis process. Estradiol is the most common estrogen hormone while others include estriol and estrone. Estradiol has also an important role in the male reproductive system by stimulating spermatogenesis [39,40]. It is present in the brain and male sex organs and its production increases at the time of sexual arousal, although overproduction of the hormone can lead to erectile dysfunction [41,42]. In females, estradiol plays a central role in the development of the reproductive organs, especially secondary sex characteristics, as discussed earlier [43].

**Figure 1:**
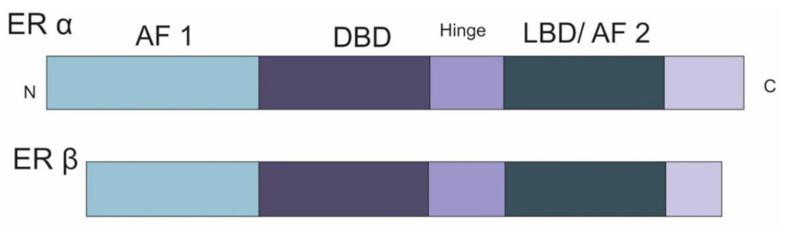
Schematic presentation of the arrangement of domains in estrogen receptors (ER) alpha and beta. N: Amino Terminal, C: Carboxyl Terminal, AF: Activation Function, DBD: DNA binding Domain, LBD: Ligand binding Domain, adapted from [54].

**Figure 2:**
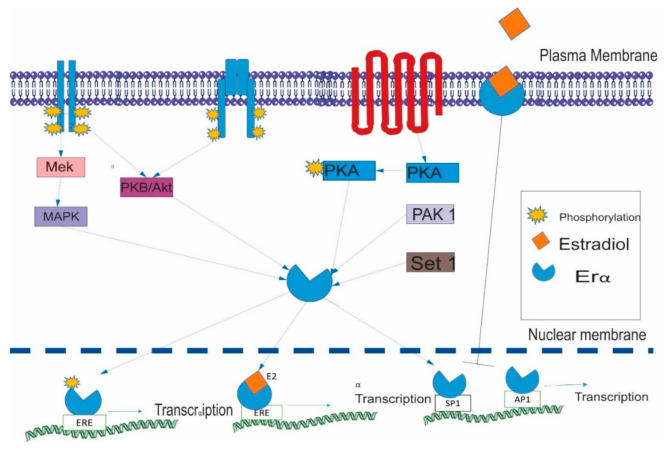
Schematic diagram showing activation of estrogen receptor alpha in both ligand dependent and independent ways. ERs can mediate transcription of target genes either by binding to estrogen response elements (ERE) or binding to other transcription factors such as SP1 and AP1 [55].

The steroidal action of estrogens on target tissues is generally mediated through two cellular pathways. One is through the nucleus and the other through the plasma membrane. In the former pathway the estrogen hormone, mainly estradiol, binds to both the ER-alpha (ERα) and ER-beta (ERβ). Both ERα and ERβ have an amino-terminal group responsible for ligand-independent activation of transcription, a DNA binding domain (DBD) in the center and a ligand binding domain (LBD) at the carboxyl end [51] (Figure 1). After binding to the ligand, the receptors translocate inside the nucleus and bind to specific transcription elements termed as estrogen response elements (ERE), ultimately starting the transcription of target genes [52] (Figure 2). The second pathway is more indirect and is called the membrane-mediated pathway, in which the ligand acts through membrane-bound ER or G-Protein-coupled E2 receptors. This interaction triggers downstream signaling via second messengers activated through the epidermal growth factor receptor (EGFR), insulin-like growth factor receptor (IGFR) and G protein-coupled receptors (GPCRs) [53]. In this case, the underlying proteins such as nuclear factor-kB (NF-kB) or Activator Protein 1 (AP-1) exert their action either by recruiting coactivators or by establishing protein-DNA complexes. In a ligand-independent pathway, the second messengers phosphorylate ER, which initiate the transcription of target genes (Figure 2). These kinases in estrogen pathways are MAPK, PKA, Akt, Erk and PAK.

**Figure 3:**
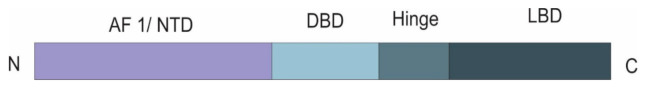
Schematic presentation of sequence of domains in androgen receptor. N: amino terminal, C: carboxyl end, AF 1: Activation function 1, NTD: N terminal Domain, DBD: DNA binding domain, LBD: Ligand Binding Domain.

**Figure 4:**
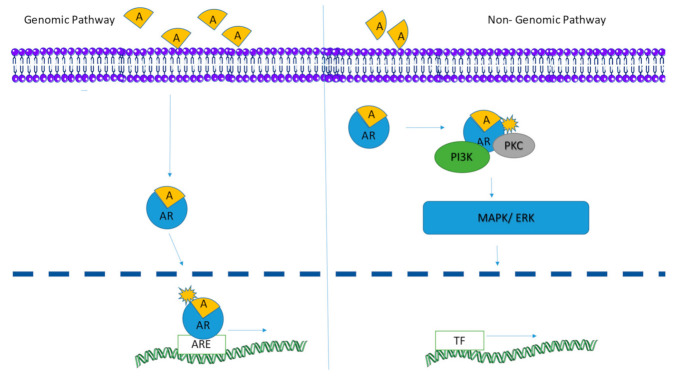
Schematic diagram showing the activation of the androgen receptor in both genomic and non-genomic pathways (after [57]).

Androgens also belong to the steroid superfamily and are mainly involved in gonadal development. Testosterone is the most common hormone, generally considered as the male sex hormone, while others include dihydrotestosterone (DHT) and androstenedione. These are vital for the development of the male reproductive system. Androgens are present in both males and females, but they differ in quantities. Like ER, AR also have an N terminal domain containing phosphorylation sites for the activation of receptors, a C terminal containing a ligand binding domain and a central DNA binding domain (Figure 3). At resting state, the receptors are bound to heat shock proteins (HSP) to avoid degradation. The binding of the ligand phosphorylates the receptor causing a conformational change and hence the release of HSP. The receptor–ligand complex is translocated to the nucleus where it binds to androgen response elements to initiate transcription of target genes [56]. Figure 4 shows the activation of the androgen receptor in both genomic and non-genomic pathways (after [57]).

BPA (CH_3_)_2_C(C_6_H_4_OH)_2_, a synthetic organic compound containing two hydroxyphenyl groups, is extensively used by the plastics industry in the production of bottles, pipes, sports equipment, CDs, DVDs and many other consumer products. As the structure of this compound mimics the estrogen hormone estradiol, extensive scientific research has observed a negative impact of BPA on human and animal health. Exposure to BPA is associated with severe endocrine-related abnormalities in both humans and animals [58]. Based on published studies, governments now implement a maximum limit of BPA use in industry [59]. However, as evident from recent studies, even lower concentrations of this compound can elicit a wide range of health problems including male and female infertility not only from direct exposure, but also by parental transfer [60,61,62,63,64]. Nevertheless, the cause–effect relationship cannot be established due to the cross-sectional design of the studies as well as the large spontaneous between- and within-subject variability of semen parameters [65]. The best evidence of an adverse effect of BPA on male fertility would be provided by prospective studies on clinically relevant endpoints, including natural or medically assisted pregnancies among men either with different exposure degrees (occupational/environmental) or with different clinical conditions (fertile/subfertile) [65].

**Figure 5:**
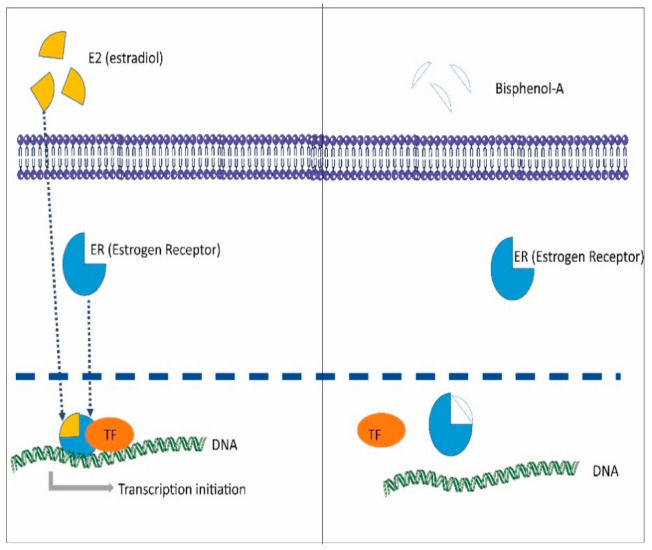
Possible mechanism adapted by bisphenol A (BPA) to cause antagonistic effects to disrupt normal signaling of estradiol via estrogen receptors.

The exact mechanisms of BPA-mediated effects in reproduction are not fully understood; however, the environmental exposure to BPA—especially in the fetal and neonatal periods—deserves attention to preserve the reproductive ability in both sexes and to reduce the epigenetic risk for the offspring [62,66]. BPA mimics the estrogenic activities of steroid hormone 17β-estradiol (E2). There are many studies confirming the negative impact of BPA on the reproductive system, especially developmental defects, which include deformed sex organs after maternal exposure to the chemical. While transgenerational effects of BPA predominantly affect male offspring, adult exposure to the chemical can cause abnormal functioning of ovaries leading to deformed egg cells [67]. In males, it can also negatively impact the process of spermatogenesis [68]. Despite being structurally similar, BPA has much less affinity for ER, although it is still able to induce the estrogenic effects at very low concentrations (0.05 mg/kg/BW) by acting through other receptors of the estrogen pathway and causing irreversible reproductive changes [69]. One possible route of action is through the estrogen-related receptor γ (ERRγ). BPA binds to ERRγ by forming hydrogen bonds with Glu 275, Arg 316 and Asn 346 in the ligand binding domain [70]. Computer-aided studies also suggested strong binding affinity of BPA to the ER (Figure 5) [71].

Other possible receptors used by BPA include G protein-coupled receptors (GPCR) and GPR30. At very low doses BPA (10−9 to 10−12 M) can act through GPCR to activate PKA and PKG to activate proliferation and differentiation of germ cells [72]. In a similar way, it can mediate its action through GPR 30 related MAPK signaling to induce apoptosis in many cells including spermatocytes [73]. In addition to strong estrogenic activities, BPA can also bind to AR and progesterone receptors, which play important roles in the development of the female reproductive tract and maintaining pregnancy. In silico analysis found Leu-701, 704, Asn-705, Met-742 and Phe-764 as being important for binding of BPA to AR, while progesterone receptors Leu-715, 718, Met-756 and 759 were found to have a stronger binding affinity, causing dysfunction in this pathway [74]. Most of the interactions between BPA and receptor residues are hydrophobic. NMR studies have provided more insights into the binding of BPA to AR, suggesting that binding occurs at N terminal domain that is present in the form of amyloid fibers [75].

Dichlorodiphenyltrichloroethane (DDT) is an organochlorine compound used extensively in the past as an insecticide in agriculture and to control malaria. The production and use of DDT is strictly restricted by an international agreement known as the Stockholm Convention on Persistent Organic Pollutants [76]. However, the convention has given an exemption for the production and public health use of DDT for indoor application to vector-borne diseases, mainly because of the absence of equally effective and efficient alternatives [77]. Also, due to its persistent nature, it is still present in the environment in the form of its metabolites, especially DDE. DDT has the ability to bind to nuclear ER and activate transcription, hence causing defects in cell cycle leading to reproductive defects. In addition to its binding to ER, it can also bind to other receptors such as seven transmembrane G protein-coupled receptors, which can ultimately activate estrogen signaling in ER-negative cells in ligand-independent manner. The isomers o,p’-DDT, p,p’-DDE have been shown to have high affinities to E2 at a concentration of 10µM [78]. DDT also shows strong binding affinity to helix 12-Ligand binding domain of ERα, causing confrontational changes [79]. Varying reports on DDT as being one of the causal agents of male infertility are evident in the scientific literature. Some suggest a positive correlation, while others do not show any correlation between DDT exposure and infertility [80,81]. DDT exposure has shown to reduce both sperm count and motility and shown to affect morphology, which are the main indicators of fertility in men [82]. DDT also affects the gonadal development in Oryzias Latipes by affecting the expression of genes for vitellogenesis VTG-1 and VTG-2 [83]. Recent use has been reported in some studies despite being banned since 1972 [84].

PCBs with the general formula C_12_H_10_−xCl_x_ are halogen-containing lipophilic hydrocarbon aromatic compounds. In the past, these compounds were largely used in electrical appliances as coolants and dielectrics, as well as plasticizers. Due to mounting evidence of the hazardous nature of the compounds on human health, their use has declined, but they are still in use in some quantities in underdeveloped countries such as Tanzania [85]. Also, due to their long half-lives, these compounds are still persistent in the environment, e.g., in air, water, sediments and in the bodies of living organisms [86]. PCBs act as ligands of ER and activate estrogenic activity by binding through hydrogen bonding and Van der Waals forces to ligand bind domain in the receptor. In silico and in vitro studies found that binding at Histidine 524 residue through hydrogen bonding with ligand inside the binding pocket is responsible for the receptor activity [87]. Many case-control studies conducted in different countries found higher average concentrations of PCBs in patients suffering from infertility as compared to healthy individuals. These EDCs affect semen parameters such as sperm motility and number and were found to be negatively associated with testosterone levels in men [88]. Animal studies have also shown similar effects of PCBs on sperm motility and number [89]. High levels of PCB 52 and PCB 180 were found in the adipose tissue of fertile and infertile men, suggesting their participation in the disease [90]. Maternal exposure to PCBs can also cause a wide range of developmental and reproductive disorders in male newborns, such as cryptorchidism (undescended testes) and hypospadias [91]. Like in males, PCBs can also cause a wide array of disorders in the female reproductive system, especially by affecting ovaries [92]. Transgenerational effects have also been observed, suggesting delayed pregnancy outcomes in females if mothers are exposed to these chemicals [93]. Long-term exposure to PCBs in female zebrafish resulted in lower number of fertilized eggs and poorly fertilized eggs due to changes in ovarian morphology [93].

Phthalates are esters of phthalic acid (C_8_H_4_O_4_) extensively used in many industrial products, especially as plastic additives. Other industries include cosmetics, household products and medical devices. A number of epidemiological studies have shown high levels of these compounds in body fluids [21]. Phthalates have the ability to bind ERα, ERβ and AR, being able to stimulate or inhibit ER but showing the only inhibitory effect on AR [94]. In most instances, phthalates demonstrate high antisteroidal activity through AR, the most important amino acid residues being Phe 764, Leu 704, Asn 705 and Thr 877 in the ligand binding domain of AR [95]. Phthalates have shown to induce apoptosis in testicular cells via an inhibitory action on AR [96]. Binding to AR involves hydrophobic interactions. Mono and dibutyl phthalate exposure can lead to AR inhibition in Leydig cells at the concentration of 50 nM and 50 µM respectively resulting in the inhibition of steroidogenesis [97]. Another study on rats showed that phthalates reduce the production of testosterone and estradiol in males, while the reduction in expression of steroidogenesis genes steroidogenic factor-1 (SF-1) and specific protein-1 (Sp-1) was also observed [98]. Transgenerational exposure affects both the male and female fetus, with female fetuses having abnormal ovarian development, increased uterine size and reduced fertility [99].

### 3.1. Effects of EDCs Acting on AR and/or ER

#### 3.1.1. Chromosomal Aberrations

Chromosomal aneuploidy (any change in structure or number of chromosomes) in gamete cells is one of the major causes of sterility and developmental defects in the offspring. The syndromes that involve sex chromosomes include Klinefelter’s syndrome (47, XXY or in some cases 48, XXXY; 49, XXXXY) and Turner Syndrome (a defected or missing X chromosome in women). Estrogen pathways predominantly control chromosomal segregation and microtubule assembly during meiosis—a major target to EDCs [100]. Exposure to EDCs leading to aneuploidy in germ cells not only causes developmental defects and infertility in offspring, but also leads to miscarriages and infertility of sperm cells [101,102]. BPA has been shown to cause defects in the meiosis process in mice and *Caenorhabditis elegans*. Rat seminiferous tubule culture was given BPA and different genes involved in meiosis were studied. Results showed defects in chromosome synapsis, which is a major cause of chromosomal aneuploidy [103]. A similar study on female mice showed similar results [104]. Another study on *Caenorhabditis elegans* showed that BPA can increase sterility by causing defects in chromosome synapsis during meiosis and it also disrupts the normal mechanism of double-stranded break repair during the process of cell division, which ultimately resulted in impaired gametes with chromosomal aneuploidy [105]. Studies on MCF-7 cell lines confirm the cytogenetic effects of BPA in both ER-dependent and independent ways at very low concentrations (0.4 μg/mL) [106]. These and many other studies suggest that BPA is a strong clastogenic agent, in most cases through disruption of the estrogen pathway. Exposure to PCBs and DDTs can also cause chromosomal defects as depicted by one study in which serum concentrations of PCBs and DDTs in infertile men showed a positive correlation with chromosomal disomy [107]. A similar study on 90 Faroese men showed similar results [108]. The presence of perfluorinated compounds (PFCs) in whole blood and seminal plasma in infertile men was found to be directly related to chromosomal disomy and DNA dimer fragmentation [109].

#### 3.1.2. DNA Damage

Despite having efficient DNA repair mechanisms, many cells are prone to DNA damage (frequently referred to as nicks or DNA strand breaks), especially sperm cells. In spermatozoids, DNA damage results in defects of the normal functionality and it is primarily caused by oxidative stress [110] and by also affecting DNA methylation [111]. Reactive oxygen species (ROS) not only cause defects in the fertilizing ability of sperm, but also induce developmental defects in children conceived by defective sperms. Egg cells have the ability to repair DNA damage in the sperm DNA after fertilization, but on a limited scale [112]. Estrogenic and androgenic disruptors such as DDTs, PCBs and BPA also cause DNA damage owing to ROS causing developmental defects in the male reproductive axis, leading to infertility [113]. BPA causes DNA damage by increasing the intracellular ROS concentrations and hence producing DNA breaks and increasing DNA migration towards tails from the nucleus [114]. BPA has shown to increase DNA damage in sperm cells and defective spermiogenesis, but human studies are missing. Epidemiological studies suggest a relationship between DNA damage and BPA concentration in human urine samples [114]. Like BPA, DDTs have also been shown to cause DNA damage [114]. Estrogens are involved in the protection of cells against DNA damage by inhibiting the production of ROS [115]. Estrogen’s vital role in the protection of DNA can be inhibited by estrogenic compounds, hence making cells more prone to DNA damage.

#### 3.1.3. Epigenetic Modifications

The role of the genetic makeup of an organism in determining its phenotype is a well-known fact, but it fails to explain the adaptive traits in response to certain environmental stimuli. Epigenetics successfully explains the effects of environment on the structure and function without changing the gene sequence [116]. The most common epigenetic modifications include methylation, histone modifications and non-coding RNAs. Environmental agents can have both a positive and a negative influence on organism survival by interfering with its epigenome either by producing offspring that are resistant to a certain environmental agent or, in the latter case, by inheritance of defects caused by paternal exposure. Recent studies are focused on transgenerational effects of EDCs on epigenome and subsequent defects in upcoming progenies [117]. Most of the studies at present are animal-based studies, often using rats as a model organism. Some case-control studies are also available through monitoring the maternal exposure and its subsequent effects on the fetus. EDCs can impact the reproductive system by either epigenetic modification or chromosomal abnormalities. Both male and female reproductive systems can be disrupted via paternal exposure to EDCs [118,119].

#### 3.1.4. Methylation

DNA methylation occurs at the cytosine residue where a methyl group attaches itself to CpG dinucleotide [120]. Certain chemicals have been reported to affect the methylation pattern in animals and hence were found to cause heritable defects to the offspring [121]. One such chemical is diethylhexyl phthalate (DEHP), which is the most common phthalate used as a plasticizer. A study conducted in rats found that DEHP can damage the male reproduction system by affecting the expression of DNA methyltransferase enzyme, resulting in varied inheritable methylation patterns, which lead to undescended testes in the offspring [122]. The effects of DEHP were tested by direct exposure to embryos and gametes and showed to alter the methylation pattern in offspring, resulting in defects in various pathways including adipogenesis, embryonic development and larval body length [123].

The effect of BPA on global and local methylation has been extensively studied. As it mimics estradiol, activation of ER alpha or beta affects the methyltransferase activity and evidence suggests their role in male fertility by affecting the process of spermatogenesis [124]. Any ER-beta agonist can decrease the activity of the enzyme, hence decreasing the extent of methylation in spermatozoa. One study showed the extent of hypomethylation as a result of BPA as an ER-beta agonist, both in a general and localized manner. It caused hypomethylation at an H19 locus and overall male infertility in rats [121]. Another study conducted in zebrafish confirmed the effects of BPA on the expression of DNA methyltransferase 1(dnmt1) and showed an overall reduction in global methylation in testes and ovaries at very low levels of exposure (1 mg/L) [125].

A recent study reported the effect of BPA on global methylation and expression of various genes related to fertility on testes and ovaries of the rare minnow *Gobiocypris rarus*. The results showed a marked increase in global methylation in both gonads and suppressed methylation in ovarian Cyp19a1a, which is responsible for the conversion of androgen to estrogen [126]. Its specific effects on spermatocytes also confirm the increase in the expression of methyltransferase and hence global hypermethylation at several loci, including myosin binding proteins and protein kinases, which are regulated by methylation [127]. Exposure of the fetus to BPA can also result in altered DNA methylation and lead to developmental defects. Maternal exposure of BPA to the fetus can cause defects in the endocrine system. Recent studies found a correlation between maternal exposure to BPA and altered methylation patterns in fetal liver. The authors suggested a positive relation between BPA concentration and DNA methylation at CpG islands that resulted in altered gene expression [128], while others suggested an increase in global methylation in tissue-specific manner [129]. Another study found 1251 differentially methylated transposon regions in humans as a result of exposure to BPA [130]. Many other compounds reported to affect the methylation pattern in offspring are mentioned in Table 2.

Other EDCs, such as pesticides, can also cause differential methylation signatures in the coming generations, hence increasing the susceptibility to disease not only in the exposed individuals but also in the next generations through epigenetic inheritance. One such chemical is methoxychlor, which has been shown to cause epimutations in sperm that can then be transferred to the offspring resulting in ovarian defects and obesity in females, primarily caused by differentially methylated regions caused by methoxychlor in the parents [143]. Phthalate BBP has been shown to alter the methylation at the promoter site of the estrogen receptor (ESR1) resulting in aberrant expression of mRNA [144].

#### 3.1.5. Histone Modifications

Histones represent a family of positively charged proteins packed inside the DNA. A histone octamer containing two of each H2A, H2B, H3 and H4 is wrapped by 146 bases of DNA termed a nucleosome, which provides the basic DNA packaging unit [145]. Post-translational modifications at the tail end of histones act as signals for DNA transcription machinery to bind, also termed as histone code [146]. Most common histone modifications include acetylation, methylation, phosphorylation and ubiquitination [147]. Alterations in histone code can lead to aberrations in gene expression. Evidence suggests a role of several EDCs in causing changes in histone code by bringing differential histone modifications.

Regulation of histone methyltransferase by enhancer of zeste homologue (EZH2) via ER is a well-established phenomenon. Xenoestrogens such as diethylstilbestrol (DES) have the capability to induce phosphorylation of EZH2, resulting in decreased trimethylation at lysine 27 Histone 3 via P13K/AKT pathway [148]. Other xenoestrogens such as BPA and Genistein showed similar results for the aforementioned genes, resulting in decreased H3 methylation, causing uterine carcinomas [149]. DES has also been shown to cause a negative impact on steroidogenesis of Leydig cells by causing deacetylation in P450scc promoter site and hence reducing mRNA expression of the gene [150]. BPA and DES can induce binding of MLL-Histone methylase 1 and 3 to HOTAIR (long noncoding RNA) promoter by histone methylation and acetylation, resulting in gene activation [151]. Exposure to BPA can reprogram the development of ovaries epigenetically by altering the expression of Histone modifying enzyme in an estrogen-dependent manner. Similarly, high levels of BPA were observed in women suffering from polycystic ovarian syndrome [152].

PCBs act through AR to alter the histone code by decreasing H4K16Ac and H3K4me3 in rats, affecting Jarid1b and SirtT1 enzymes, and decreasing AR [153]. Many other studies confirm the ligand binding of PCBs to the AR, changing the histone code, as it can activate the transcriptional activity of AR by its action of demethylase Jadrib1b, which catalyzes removal of trimethylation at H3K4me3 [154]. Exposure of *Xenopus laevis* to Ioxynil (IOX) and tetrabromobisphenol (TBBP) was found to suppress tri and tetra methylation at H3K4 by suppressing the expression of TH receptor beta [155].

#### 3.1.6. Micro RNAs

Micro RNAs (miRNAs) are a family of non-coding RNAs usually comprised of 18–25 nucleotides, which have regulatory action on gene transcription [156]. Recent studies suggest that miRNAs have an important role to play during development, as an altered miRNA profile can lead to developmental defects in both the male and female reproductive tract [157,158]. Altered expression of miRNAs in the semen of infertile men compared to fertile ones has also been observed, suggesting their use as potential biomarkers for idiopathic infertility [159]. Several studies related to miRNAs expression during spermatogenesis confirm their role in the regulation of the process [160]. EDCs have also been shown to affect the expression of miRNAs [161]. Chronic exposure to EDCs mixture in human males may potentially lead to spermatogenic failure through changes in miRNA expression, which could post-transcriptionally dysregulate mRNA targets that encode proteins participating in cell death in testicular cells [162]. Distinct miRNA expression patterns during oocyte/embryo development imply important regulatory roles of miRNAs, while EDCs such as BPA appear to be implicated in the disruption of their expression/activity altering gene expression [163,164]. Estrogen signaling modulates the biogenesis of miRNAs such as miR-21, -155 and -124 [165]. Since BPA resembles estrogen’s action, it is possible that it interferes with the expression of multiple miRNAs but specific evidence in reproductive models incorporating oocytes/embryos is scarce [165]. An in vivo animal study reported that BPA, acting at the transcriptional level prior to processing, resulted in decreased expression of 45 different miRNAs in the fetal ovary and abnormal expression of associated target genes [166]. Another in vivo study reported that BPA dysregulates miR-224 in rat granulosa cells, which is important for cumulus cell expansion/oocyte maturation via regulation of aromatase [167]. In human breast cancer cells, BPA was reported to induce ERα signaling reduce miR-21, Let-7 and miR-15b expression while enhancing miR-638, miR-663 and miR-1915 expression [168]. In human placental cells treated with BPA, miR-146a was increased/associated with slow proliferation and higher susceptibility to toxic effects of harmful agents [163]. In human endometrial cells, BPA significantly suppressed miR-149 and increased miR-107 [169]. The mechanisms by which EDCs affect gene expression in oocytes and embryos are likely not limited to microRNAs that directly target important genes but may also involve microRNAs that participate in epigenetic control [170]. Like all estrogen-dependent pathways, BPA treatment may disrupt this axis disturbing downstream gene changes that control oocyte competence [164].

#### 3.1.7. EDCs, Obesity and Fertility

Exposure to ECDs leads to obesity [13] and obesity related diseases such as female and male infertility through various mechanisms [171,172]. Obesity status appears to be associated with the interaction of EDCs with fertility [23]. EDCs such as BPA phatalates and PCBs are lipophilic and bio-accumulate in the adipose tissue, resulting in exacerbation of their endocrine disrupting effects, thus, their biological effects appear to be prolonged in obese people [173]. Bioaccumulation of EDCs stored in the adipose tissue (obesogens) is greater in obese persons due to a larger fat mass that reduces the clearance rate of the chemicals over time [174]. Exposure to EDCs such as BPA and phthalates may reduce gonadotrophin secretion by promoting insulin resistance [173]. Permanent exposure to obesogens may increase aromatase activity that increases E2 levels, which in turn can compromise hypothalamic–pituitary axis downstream signaling events altering spermatogenic process [173]. DDT exposure induces transgenerational events promoting obesity and sperm epimutations [175]. There are few epidemiological studies in humans focusing on the effects of obesogens on sperm quality and therefore definite conclusions cannot be drawn on their exact interaction with molecular mechanisms involved in the production and function of human sperm [173].

Many recent studies suggested that the timing and dose of EDCs are very important for determining the phenotypic outcomes. The most critical one is the exposure of pregnant women, as many of these compounds can have an effect on the developmental process [176,177,178]. Prenatal exposure to EDCs has been reported to affect the sexual development of both male and female fetuses and is the basis of sexual disorder in adulthood [178,179,180]. Sex hormone binding globulin has the ability to bind maternal hormones and is responsible for blocking these hormones from entering the placenta, however many EDCs including BPA have a lower binding affinity to it [181]. Exposure via breastfeeding has also been reported to cause reproductive disorders in both men and women [182]. Adult exposure as a possible cause of reproductive disorders including subfertility, fertility and hormonal imbalance has been also reported [182].

## 4. Conclusions

EDCs can affect the reproductive potential of an organism in many ways. The effects can be seen in the individuals exposed or transferred to the next generation via epigenetic inheritance. Estrogens and androgens are major players in the normal growth and reproductive functioning in organisms. Any disruption in the pathways can lead to malfunctioning in both male and female reproductive systems. Almost all major classes of EDCs have the ability to target the androgen or estrogen pathways or both. EDCs can affect both pathways at both genetic and epigenetic levels. Only a few studies are available on the structural similarities/binding affinities of these compounds with natural ligands, which enable EDCs to bind receptors. To be able to devise efficient therapeutic strategies to reverse the negative impacts of EDCs on reproduction, it is recommended that all possible targets (proteins) and their impacts are studied.

## Figures and Tables

**Table 1:** The main studies showing the binding of EDCs to androgen receptors (AR) and/or estrogen receptors (ER).

EDCs	Pathway/Receptor	Antagonist/Agonist	Reference
**BPA**	ER	Agonist	[44]
	AR	Antagonist	[45]
**DDTs**	ER	Agonist	[46]
	ER	Agonist	[47]
	ER	Agonist	[44]
	AR	Antagonist	[46]
	AR	Antagonist	[47]
**PCBs**	AR	Agonist, antagonist	[48]
	ER	Agonist	[48]
	AR	Agonist	[49]
**PBDEs**	ER	Agonist, antagonist	[50]
	AR	Antagonist	[50]

**Table 2:** Differential DNA methylation caused by different classes of endocrine disrupting chemicals (EDCs).

EDC	Species	Concentration	Locus/Genes	Reference
**BPA**	Wistar Furth rat	250 μg/kg/day	transcriptional initiation site of the alpha-lactalbumin gene	[131]
	Female Wistar Rat	40 μg/kg/day	Fkbp5	[132]
	Mouse	0–80 uM	Mybph, Prkcd	[127]
	Pregnant Wistar rat	50 μg/kg/day	↓ hepatic glucokinase, ↑Gck	[133]
	Rat	10 μg/kg/day	↑ Scgb2a1	[134]
	Mouse	50 ng/kg, 50/kg μg, 50 mg/kg	Jak-2, Rxr, Rfxap, Tmem 238	[135]
	Mouse	50 μg and 50 mg/kg/day	Myh7b, Slc22a	[136]
	Rare minnow *Gobiocypris rarus*	15 μg/L	↓cyp19a1a	[137]
**Methyl mercury**	Rat	2 mg/Kg/Day	↓ DNMT (global hypomethylation)	[138]
**PCBs**	Rat	1.1 mg/kg/day	↓DNMT-1, 3a, 3b (global hypomethylation)	[138]
	Slider turtle	0–200 μg/L	Aromatase promoter	[139]
**DEHP**	Human	-	IFT140, TESC and PRDM8	[140]
	Rat	100 mg	Dnmt3a, Dnmt3b and Dnmt1	[98]
	Mouse	20 or 200 μg/kg/day	Y chromosome genes (↓ Sry; ↑Eif2s3y; ↑chromodomain protein; ↑Cdyl; ↑Zfy2)	[141]
**(DEHP in mixture with genistein)**	Rat	0.1 and 10 mg/kg/day	Kitlg, Rsk2, Nr3c1, Nqo1, Lif, Fyn, Dep-1, Gpr116, Pfn2 and Ptgr1	[142]

## Data Availability

Not Applicable.
